# Dr. Brainard’s Address before the American Dental Association

**Published:** 1865-08

**Authors:** 


DENTAL REGISTER OF THE WEST.

VOL. XIX.] AUGUST, 1865. [No. 8.

### Original Essays and Communications

### DR. BRAINARD'S ADDRESS BEFORE THE AMERICAN DENTAL ASSOCIATION.

GENTLEMEN :

It gives me great pleasure to meet here the members of so honorable and useful a profession as yours. The profession of Dentistry is one of very modern origin, and is that branch of the medical profession which owes its development and perfection most essentially to our own country, and is, indeed, I think I may say, the one branch of the profession in which we Americans can claim especially the pre-eminence. To be an American dentist is a recommendation in all the principal cities of Europe, and although the medical profession in general, and the surgical department especially, has an honorable position in the literature and among the profession in foreign countries, it can hardly be said to have a claim to the title of any pre-eminence.

I have said that your profession was of comparatively recent origin. It is almost, I think, within the memory of many persons here present, when it was regarded as a merely mechanical operation, little better than the higher branches of mechanical employment. Step by step it has developed itself to a degree that, in perfection, in usefulness, it does not, in my opinion, rank second to any of the single branches of medical or surgical science, [applause]; so that at the present day, to be without a dentist would be to be without one of the essentials of civilized life. [Renewed applause.]

Now, in speaking of its having a mechanical origin, I do not intend anything disrespectful to it. My own especial branch of the profession is surgery, and, I might say, a part of surgery; and it is not very long since surgeons, as a class, occupied a position far less honorable and important than that occupied by dentists at the present day. In the middle ages in Europe, so called, or rather in the latter part of the middle ages, from the fourteenth to the end of the fifteenth century, or thereabouts, surgical operations were performed by barbers. There was no distinction between those that performed a surgical operation and those that cut hair, and curled hair, and powdered hair. The duty of the surgeon was considered to be to perform an operation when he was directed, and as he was directed by a physician, and, when he had performed the operation, to retire and leave the case to the physician. And it happened, after a certain period of time, that these operators acquired a certain amount of knowledge beyond the immediate needs of operating, and were called upon to advise about operations, and about the treatment of cases after operations. And this was considered a great innovation upon the profession. It was considered something outrageous and disgraceful to the profession; and the man who should have consented to meet a surgeon in consultation, would have been expelled from the faculty for consulting with a quack. I have in my library a work published by the President of the Faculty of Medicine, in Paris, entitled "*The Brigandage of Surgeons*," setting forth that they were asserting claims which, if allowed, would be the destruction and disgrace of the medical profession.

This comparison I bring forward for the purpose of sustaining the assertion that I make, that the difficulties under which the dental profession labors are nothing new. By speaking of the difficulties under which you labor, I mean this: the difficulty and length of time which it has taken for the honorable, and educated, and competent men of that profession, to assert and maintain full equality in professional standing with members of the medical profession who practice other branches of it. That difficulty has existed in regard to every particular branch of the profession that has at any time been embraced by any particular class of men, and is not peculiar to the dental profession. It results from that deep seated prejudice, for I can call it by no other name, that has existed from the earliest times, and is inherent apparently in the nature of every profession, and that is to resist innovations or changes in regard to the doctrines or the practices of that profession. This peculiar aversion to changes results from the nature of professional education, and is one of those things that is treated of by Lord Bacon in his *Novum Organum*, under the name of "Idols of a Class," or those things which prevent members of a particular class from seeing the truth in regard to their own profession. It is, and has been, one of the great obstacles to the progress of the medical profession. It is an obstacle which at the present day is partially, but only partially overcome. It is the thing which has prevented a large number of men of genius and industry in the profession from embracing and following out the study and practice of a particular class of diseases, in such a way as to have perfected our knowledge of the nature and treatment of such diseases.

Now, the principle that I wish particularly to assert here is this, that the medical profession, in order to be most useful, in order to acquire its due influence over the community, in order to perfect its knowledge of the nature and treatment of diseases, must adopt a special course of study; each individual member embracing that course which he judges on the whole to be best adapted to his faculties, and leaving out to a certain extent others for which he has no qualifications. I advocate special studies and special practice; and, although the words have been somewhat discredited, I advocate "specialties" and "specialists."

Now, I undertake to say that the very great opposition which this doctrine has met in the profession, is not founded upon reason, or justified by the experience of the profession. It is an opposition which is working to the disadvantage of the profession as well as the public, and to the manifest disadvantage of a very great number of the individual members of the profession; and therefore I wish to insist a little upon the point.

What are the natural divisions of the science and practice of medicine? Is there no natural division? I hardly think that any one would be so bold as to assert that there are, and ought to be, no natural divisions. For a long time, when the science of medicine was in a very rude and imperfect state, the members of that profession did study and practice all its branches; and in the older works on Surgery, Pharmacy and Chemistry were as much treated of as the operations of Surgery. Still, even at that time, there was the commencement of division, and the first was into Pharmacy, Surgery and Obstetrics. The next division which came was into Medicine and Surgery, Surgery being, as I have stated, the mechanical application in the manner already indicated. At a later period, there commenced to be apparent the distinction between the Obstetrical department of the profession, and the separation between Pharmacy and Medicine was accomplished entirely. By degrees the distinction between obstetricians and medical practitioners came to be recognized in particular localities, in large cities especially, to a very considerable extent; so that medicine, towards the middle of the last century, might be said to be divided into Pharmacy, Medical Practice properly so called, Surgery, and Obstetrical Practice, whilst yet it remained true in regard to the greater portion of the civilized world—the country in general, in America as well as in Europe—that the larger number of practitioners continued to practice all the branches, to collect and prepare their own medicines, to practice Pharmacy, Surgery, and Obstetrics. And at the present day, this is the case with a very large part of the profession throughout the civilized world.

Now, it is manifest—all experience and reason show—that men who practice medicine in this way, practice it only in a rude and imperfect manner; that they neither understand Pharmacy, nor Medicine, nor Obstetrics; that, from the nature of things, they are incapable of acquiring skill in any one of these branches; that it is absolutely necessary, in order that there should be any progress in regard to the science or practice of medicine, that some of these should be excluded, and others proceeded with especially, by each individual member of the profession. Let us examine the question for a moment. The Dentists, as a body, have, according to my own knowledge and observation, perfected the mechanical means of performing operations beyond what has been done in any other branch of the profession. They are better mechanics than the Surgeons, and their instruments for accomplishing the different objects which they have in view are more numerous and better suited to their purpose than are the instruments of surgeons. Now this is essential to the proper performance of dental operations. And how has this happened? It has been simply that there have been dentists, as a class, who have devoted their attention to that purpose; and we as surgeons never could have invented or perfected these instruments, and consequently could never have perfected dentistry. And that division of labor is a thing which, at the present day, is manifestly necessary, and which no one now disputes.

In regard to surgical instruments, there are two departments in which they have been singularly perfected. The one is in regard to those instruments which are used for crushing urinary calculi, which are the most admirably adapted to their purpose. How did that come about? Three men of genius at the same time happened to devote their whole attention to that thing—the crushing of stone—and they especially perfected those instruments. And without that perfection of these instruments, the crushing of urinary calculi must have remained forever, as it had up to that time, a mere phantom floating in the mind, without any practical application whatever. Therefore it was necessary in this particular that there should be special studies. The other branch in which I consider the mechanical means to be wonderfully perfected, is that regarding instruments for operations upon the eye. These instruments have been brought to such a degree of perfection, of delicacy and accuracy, that they are capable of accomplishing things almost inconceivable, and accomplishing them regularly, constantly, and without difficulty or danger. Now, how has this happened? It has happened in the same way; there have been a class of men who have devoted their attention to that subject exclusively, have thought of nothing else, have worked at nothing else but the perfection of means for the accomplishment of that which they saw before them to be done. But when you come to other things that have not been made specialties, the condition of our science is singularly rude and imperfect. There is nothing in our science at the present day which has the slightest claim to be respectable apparatus for the treatment of fractures. Particular individuals, by the attention which they have paid to it, and by an excess of superior mechanical talent or ingenuity, have been able to accomplish with the instruments which they use a considerable degree of success, but there is no instrument for any given fracture that can be mentioned, that can be taken by a person of ordinary good education of the profession, and put upon the member in such a manner as to accomplish any perfect result.

The greater number of the instruments which are used in the profession for fractures of the leg, and called "fracture boxes," are no better than dry goods boxes, [applause], and simply serve to accomplish the result of concealing from the surgeon the position in which a limb may happen to lie. [Renewed applause.] What is the reason of that? The reason is, that there never has been as yet an instance in which a man devoted himself to fractures as a specialty, and nothing else, and this is the one branch in which a speciality is most needed. They are the species of accident which are the most frequent, and which disable a man more than any other, and entail untold miseries upon him if unskilfully treated ; but the instruments for the accomplishment of this purpose never will be reduced to any great perfection, until it shall be known that the devoting of time and talents to one subject leads to honor and not to being partially thrown out of the profession. [Applause.]

If I should be unfortunate enough to meet with a fracture of the jaw, the first thing I should do would be—not to send for a surgeon—but I should send for a dentist. [Cheers.] They have directed their attention to the mechanical means and apparatus necessary for holding the jaws in their place, to such an extent, that they are better qualified to make them than surgeons are as a general thing, and, perhaps I might say, more than any surgeons are.

I might go on and point out to you that with regard to no one thing about them are the instruments used by surgeons, the best adapted to their purpose,

We are disputing, at the present day, all over the world, what kind of sutures are the best to use. A great many of our instruments for the purpose of ligating arteries and performing similar operations, are singularly rude and imperfect, and their imperfection is only remedied by the skillful use of the fingers of the surgeons.

How is this to be remedied? It is to be remedied, gentlemen, by special study; by the profession changing its views upon that subject, and saying to the young men, when they are entering the profession, and when they are about to leave the schools, that it is better for them to devote themselves to some particular branch of the profession and try to understand it. I am often called upon by young men from various parts of this country, who are visiting the West for the purpose of locating themselves in their practice. They very frequently come to Chicago, and we are always glad to see them. We are very proud of our city, and if you want to get into the good graces of any Chicago man or woman, you have nothing else to do but to tell them it is a nice place. [Laughter.] And these young men come here and they say: "What kind of a place is Chicago for a professional man?" Now this is a very hard question to answer, because politeness does not permit me to ask another question. I could say to the young man, that if you know any one thing better than the generality of the profession, it is a good place for you; but if you do not, it will be a bad place for you. And for those young men who are incapable of applying their knowledge in such a way as to earn their daily bread, incapable of using their knowledge for the benefit of any particular class of men, so as to make it desirable for them to call upon them, Chicago is not the place. That is the difficulty under which members of the profession labor when they would enter into practice.

How is this to be remedied? It is to be remedied, in the first place, by acting upon public opinion. The profession which listens with leaden ears to the propositions which come from members of it to change the time honored usages to which it is subjected, is sensitively alive to intimations which come from the people who employ them. Public opinion in this country is law, and in order that the laws be made good, public opinion must be enlightened. Individuals are powerless, but ideas are irresistible; and the way to remedy it is to take the idea or fact that in order to make the profession useful and powerful, it must be developed and perfected in all its branches, constituting each one of these branches one body, each part of which co-operates in its proper sphere and most useful manner in advancing the interests of the whole profession. [Cheers.]

Now, in saying that I am in favor of special studies and practice, in that way, I do not commit myself to anything; and the profession won't regard this as anything but a "glittering generality." Therefore, I state that I think there ought to be Dentists to attend to the teeth, Oculists to attend to the eye, Aurists to attend to the ear, and special Physicians to attend to diseases of the heart and lungs, and make the physical examinations which are so difficult, and a special class who will be able to use the microscope for special examinations, that there should be not only practitioners of obstetrics, but those especially devoted to different branches, I mean that the incapable obstetrical practitioners never should be allowed to use instruments; that there should be men qualified for that. And then in regard to surgery, that it should in addition have a number of branches. That in these there should be one branch devoted especially to the treatment of fractures. That there should be another branch devoted to the treatment of tumors, without absolutely circumscribing these departments by definite lines at the present time. When that is done, then the profession will cease to occupy the principal part of the time in its meetings or associations with quarreling with quacks. The man who is thoroughly accomplished in any particular department of his profession, is very little troubled by quacks. [Applause.] Then the public will come to know the usefulness of the profession.

The dental profession, at the present time, is not consulted in one case in a hundred, or one in a thousand of those which require the care of a dentist. That is because the medical profession is not educated to the proper standard, and does not tell these people, as they ought to, that in cases of difficulties about the teeth, they should apply to a good dentist.

The same is true in regard to Surgery. There are operations enough that ought to be performed, in every populous county in the State of Illinois, and which are not performed, to give employment to all the surgeons in those counties. They do not know that there is any man who applies himself to that particular kind of disease, and when they look around for information, they look to that particular kind of advertisement in the newspapers ; and you know what kind of information they get there. [Laughter.] I repeat, then, that the way of progress in the medical profession is in the way of special studies.

How is this to be brought about? I would not have you to suppose, by any means, that there should be a special school for every department of medicine and surgery. On the contrary, I would very much prefer that there should be no divisions whatever. And if I might be permitted to express an opinion upon a subject which may be delicate, it would be an opinion with regard to the dental profession, that they had better not be separated from the medical profession. I think it would be better for them to receive their education in the same schools and to the same extent as other members of the profession. And I think that in order to effect their education, there ought to be, and will be, perhaps not in my day, but there will be professorships of diseases of the teeth in every respectable medical school in Christendom. [Cheers.] What is taught at the present time in most medical schools in regard to teeth, is the order and time of dentition ; and then, in case the child is sick during that period, that the gums are to be lanced. [Laughter.] In some schools, there is a little advance upon that.

But there is no medical school, so far as I have any knowledge, where the diseases of the teeth and the causes which produce them, the means of obviating them, the irregularities of the teeth, and the means of correcting them; the best thing to do in case of any particular appearances of the teeth; I am not aware that in any institution, even to that extent, it is properly taught.

Of course there must be colleges of dental surgery, so long as dental surgery cannot be learned elsewhere. This is a want, and until we can supply it, the practice of surgery must be necessarily more or less imperfect. It is necessary, in my opinion, that the dentist should he an educated physician. [Cheers.] It is necessary that he should understand the structure of the body beyond the teeth. [Cheers.] And I will mention this curious thing about anatomy and quackery. Anatomy in itself would not seem to teach a man much of a practical nature; but I have never yet seen an accomplished anatomist who was a quack, or a quack who was an anatomist. There is an incompatibility between the pursuit of that sublime science, which makes the two incompatible; and if you will fetch before me any man, whether he be a dentist or otherwise, and he will tell me all that is known in reference to anatomy, I will accept that man as a scientific man, without asking him another question. [Laughter and applause.] That is the foundation of all medical science, and therefore you must have that; and you must have, in addition to that, the knowledge of physiology. You must have an especial knowledge of the action of medicines, as they operate upon the human system, not to speak of those other branches of science and accomplishments, outside the profession strictly so-called, which are so necessary to give influence to science, to render man happy, to adorn his life, and make him a gentleman. I would have, in the first place, all the different classes of the medical profession educated in the same schools, to the extent of acquiring this general knowledge of which I have just spoken; and then I would have in each college not only a professor of diseases of the teeth, but I would have a special professor with regard to a certain number of other branches, at present of the diseases of the eye and ear, and of the nature and treatment of deformities of every kind. These branches have all acquired a degree of development which requires them to be treated from separate chairs ; and when the student had got sufficiently accomplished in general principles, I would have him adopt that branch which he proposes to follow, and devote his special attention to it ; and I would have these new chairs instituted from time to time as the wants of the community seem to require.

Medical science, which two hundred years ago was imperfect, has at the present time acquired a degree of development which renders it impossible that any one should master it in all its details. No intelligent man devoting his time to it can read all the works connected with it, that appear in the English, German and French language, from one end of the year to the other. It is a physical impossibility. It is a physical labor that he could not endure. How then is he to become acquainted with the details of all the different branches, able to perform every kind of operation, able to prescribe for every kind of disease ? In proportion as the science advances, it will become still more extended and difficult. As each science is perfected, this is the rule. There will be future advances, which at the present day it will be unwise, if not impracticable, to define.

I have said that the profession, organized in the manner in which I propose, should be formed in parts not in conflict with each other, but should constitute one harmonious whole. The dentists are capable of exercising a great influence upon society. They are a numerous, enlightened, and I am happy to say, wealthy and influential body of men, and we, with all our prejudices, cannot afford to leave them separate and standing off. [Laughter.] The physicians and surgeons are capable of exercising a great influence in favor of the dentists, and they would, if better informed and more enlightened, exercise a greater influence than they do, by directing their patients always to apply to them for advice with reference to every question which might arise as to the teeth. This would be an advantage to the dental profession, and they would get it to a much greater extent if they were a recognized part of the medical profession, as they deserve to be. So with all the other different specialties ; and whenever I see a man taking up one of these specialties, and for years together undertaking the labor connected with it, I say, " Go ahead, do what you can in that branch of the profession." I do not regard him as in any degree conflicting with my own or any other branch of the profession ; I am happy to see it, and I carry my approval to the extent of recognizing the principle in branches of the profession of which I might have, myself, some little doubt.—Such branches as the application of electricity to diseases, about which our information is so imperfect and indefinite. I consider that the application of electricity, by an intelligent man, would probably be of great use, and therefore when a man undertakes that specialty, I think he enters upon a useful work, and one that it is necessary to make a specialty of before it can be relied upon by the profession generally. It is the same with regard to another class of practitioners—the movement-cure men. That term—" Swedish movement cure"—is an unfortunate name. The method of treatment to which it is applied, did not originate in Sweden, but is founded upon the principles of physiology, justified by experience, incapable of being applied by practitioners who have not the necessary knowledge, and promising, in the future, as it shall be developed, the relief of a large class of diseases which have been, heretofore, and which are, at the present time, to a great extent, beyond the reach of surgical treatment.

These, gentlemen, are the essential outlines of the ideas which I wished to present to you. " A word to the wise is sufficient," and therefore I need not dwell upon them. It has given me great pleasure to know that this association is a successful association, and that it is attended by a large number of men from various and distant parts of the country. You have already received a welcome from your own profession here: that welcome was only the expression of the feelings of the general community with regard to yourselves and every other body which comes here for purposes so useful and honorable as yours, and therefore, from the harmony of your session, I have only to hope that it may be useful, interesting and improving, and if you ever need a place to meet in on another occasion, I would take the opportunity of asking you, on behalf of the medical profession and our citizens, to come back to Chicago. [Loud cheers.]

Dr. Chesebrough, of Toledo, moved:

That the thanks of the association be unanimously voted to Professor Brainard, of the Rush Medical College, for his assistance to us in our course of life, and that the sense of the convention be expressed by rising.

Professor McQuillen, of Philadelphia, moved as an amendmend:

"And that the learned Professor be requested to furnish a copy of his Address for publication in pamphlet form."

Dr. Chesebrough accepted the amendment, and the motion, as amended, was adopted.—*Chicago Medical Journal.*

